# Diffuse peritonitis secondary to urachal cyst abscess in a postpartum patient

**DOI:** 10.1016/j.ijscr.2024.109584

**Published:** 2024-03-31

**Authors:** Martina Bertoni, Armando Pintucci, Anna Locatelli, Angelo Miranda

**Affiliations:** a School of Medicine and Surgery, University of Milano Bicocca, Italy; b Obstetrics and Gynecology, Pio XI Hospital, Desio, ASST Brianza, Italy; c Obstetrics Unit, IRCCS Fondazione San Gerardo dei Tintori, Monza, Italy; d General Surgery, Pio XI Hospital, Desio, ASST Brianza, Italy

**Keywords:** Case report, Urachus, Cyst, Abscess, Infection, Pregnancy

## Abstract

**Introduction and importance:**

Urachal cyst infections during pregnancy are exceptionally rare, posing diagnostic challenges. This case report contributes to the limited literature, emphasizing the rarity, diagnostic difficulties, and the need for heightened healthcare provider awareness for timely intervention.

**Presentation of case:**

A 32-year-old pregnant woman with persistent pelvic pain, fever, and urinary symptoms sought care with inconclusive initial diagnoses despite multiple ER visits. Labor revealed a palpable mass, and postpartum, a CT scan identified a urachal cyst abscess. Urgent laparoscopy confirmed peritonitis, leading to cyst removal, antibiotics, and a subsequent laparotomy. Histology confirmed an abscessed urachal cyst.

**Discussion:**

Urachal cyst infections in pregnancy, exceptionally rare and diagnostically challenging, highlight the importance of considering them in abdominal pain differentials. Diagnostic tools, such as ultrasound and CT scans, can be misleading, emphasizing the necessity for a multidisciplinary approach.

**Conclusion:**

This case report underscores the challenges in diagnosing and managing an infected urachal cyst during pregnancy, stressing the need for awareness and a comprehensive diagnostic approach for optimal outcomes. The rarity of such cases warrants increased attention within the medical community.

## Highlights

• **Rare Presentation**: Uncommon infected urachal cyst during full-term pregnancy, challenging diagnosis
• **Distinctive Clinical Features**: Afebrile patient with palpable mass; initial misdiagnosis as colliquated fibroid
• **Diagnostic Challenges**: Rare urachal cyst infection; atypical symptoms, delayed diagnosis in pregnancy
• **Multidisciplinary Approach**: Successful management with obstetric, surgical, and infectious disease collaboration

## 1 Introduction

In normal embryological development, the urachus is a tubular structure that connects the urogenital sinus and the allantois. The urachus begins to obliterate during the fourth and fifth months of gestation. This narrow epithelial tube progressively transforms into a fibromuscular structure as the bladder descends into the pelvis. At some point during or after gestation, the urachus obliterates to form the median umbilical ligament. The exact timing of urachal obliteration remains controversial. Bartholomaeus Cabrolius first described urachal persistence in 1550 [1]. Partial or complete failure of urachal obliteration can give rise to four distinct embryological malformations: patent urachus, umbilical-urachal sinus, urachal cyst, and vesico-urachal diverticulum [1,2]. Based on autopsy studies, a urachal residue can be found in as many as 2 % of the general population, remaining asymptomatic throughout life, occasionally diagnosed incidentally during surgical procedures or radiological studies, and only rarely symptomatic [3]. There are very few clinical cases of urachal cyst infection in pregnant women in the literature. Physiological changes that occur during pregnancy, associated with bacterial growth, often originating from the urinal tract, likely play a significant role in making urachal anomalies symptomatic. Our experience may be helpful for the complex diagnosis of this condition.

The work has been reported in line with the SCARE criteria [4].

## 2 Presentation of case

D.S.R. is a 32-year-old woman of Caucasian ethnicity, gravida 1 para 0, with no medical history, no history of intra-abdominal surgery and no prior urological disease. Her pregnancy was uneventful and was under the care of a private physician. Between August 23, 2023, and September 9, 2023, from 35 + 4 to 37 + 6 weeks of gestation, she visited the Emergency Room three times in four days due to persistent pelvic pain, occasionally worsening, associated with fever at home (maximum 37.8 °C), abundant leukorrhea, sensation of incomplete bladder emptying and dysuria. These symptoms were accompanied by sporadic uterine contractions. At the obstetric evaluations performed in the Emergency Room no clinically relevant condition was diagnosed and the uterine contractions were not significant. She was initially prescribed antipyretics, urine culture (which the patient never performed), and empirically an antibiotic therapy (Cefixime 400 mg for 5 days, followed by Amoxicillin/Clavulanic Acid 1 g three times daily for 5 days), but her symptoms did not improve.

During her last visit on September 11, 2023, at 38 + 1 weeks of gestation, her blood tests showed a white blood cell count of 16,300 g/dL and a C-reactive protein (CRP) level of 158 mg/L, even though she was afebrile. Her obstetric examination revealed a posterior cervix, partially effaced, and dilated 1–2 cm, with a cephalic presentation, level − 4. Consequently, she was admitted to the hospital for prodromal labor and a suspected urinary tract infection. A urine culture was sent for analysis and a broad-spectrum antibiotic therapy with Amoxicillin-Clavulanic acid 1 g IV three times a day was administrated in continuation of home antibiotic therapy.

**Fig. 1:**
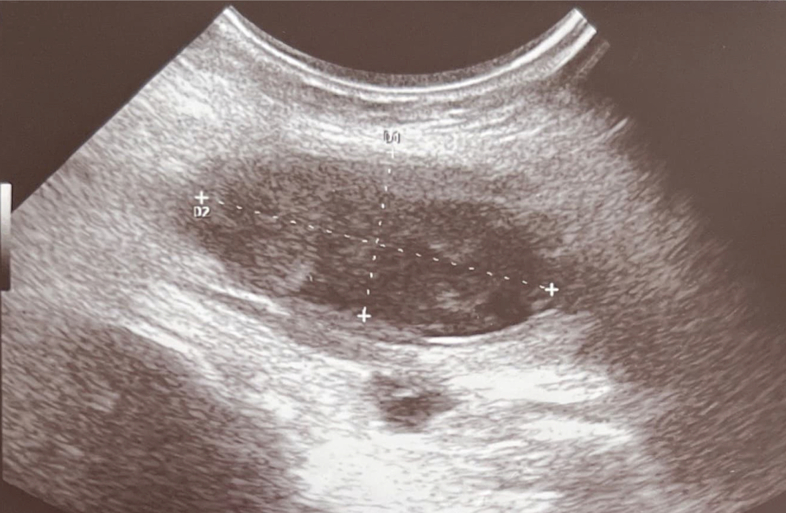
Abdominal ultrasonographic findings (longitudinal view) of a suprapubic and supravesical lesion during labor, initially considered as a colliquated myoma.

Active labor began spontaneously after nine hours, and the patient requested pain relief through an epidural catheter. However, she continued to experience severe suprapubic pain. During labor on physical examination, a 5 cm soft and painful mass was palpated in the suprapubic region. A transabdominal ultrasound during labor revealed a non-homogeneous image with clear margins measuring 2 × 6 × 4 cm, that was considered suggestive of a colliquated median anterior isthmic subserous myoma [Fig. 1].

On September 12, 2023, an operative delivery was performed using a Kiwi suction cup due to meconium-stained amniotic fluid, prolonged second stage of labor, and maternal exhaustion. A paramedian episiotomy was carried out and sutured continuously. Estimated blood loss was 400 mL. During labor the maximum body temperature was 37 °C. Placental swabs were sent and showed positivity for S. Homini whereas the urine culture was negative.

The patient referred symptom improvement on the first day of the postpartum period and she had normal vital signs. However, her CRP level worsened, reaching 171 mg/L, despite a negative urine culture. Ongoing antibiotic therapy was continued.

On the second day of the postpartum period, at 10:30 PM, the patient complained of severe suprapubic pain. No nausea or vomiting were present, and the bowel was patent. The vital signs were normal, and CRP levels decreasing. Abdominal examination revealed tension, with a weakly positive Blumberg sign. A transabdominal ultrasound confirmed the presence of a mass of 56 × 38 × 47 mm interpreted as median anterior subserous myoma with peripheral vascularization

[Fig. 2].

**Fig. 2:**
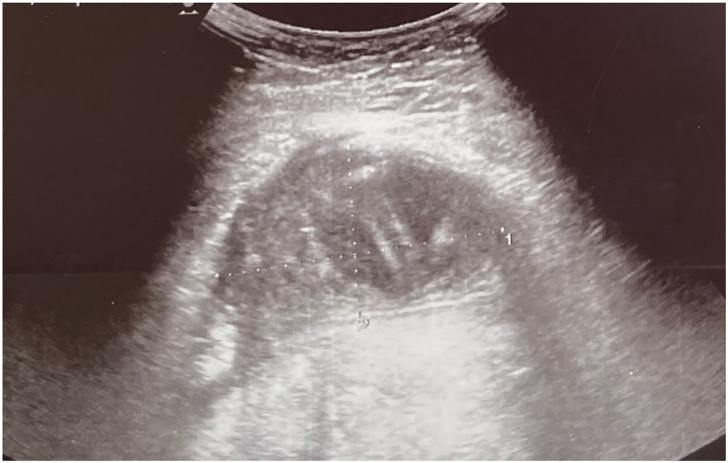
Image consistent with a typical uterine fibroid: a well-circumscribed, round, solid lesion with iso-hyperechoic texture, well-defined margins, displaying the characteristic fibrous appearance and peripheral vascularity.

**Fig. 3:**
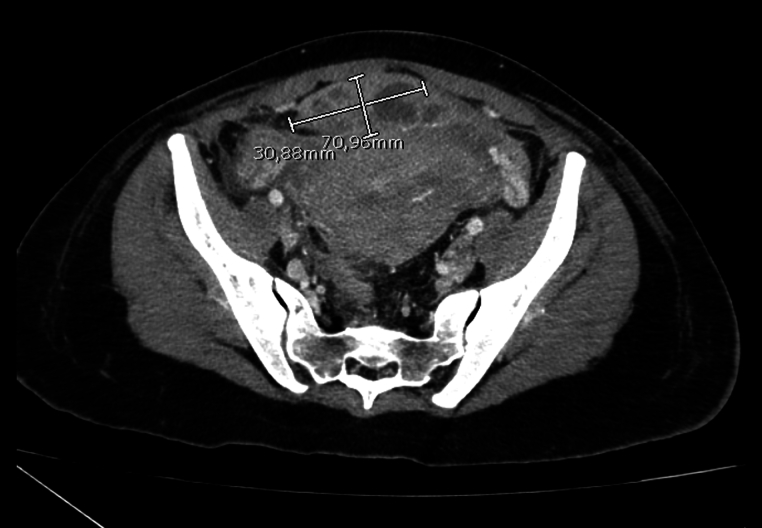
Coronal view of the CT abdomen and pelvis with IV contrast performed demonstrating an oval alteration within the medical umbilical ligament anterior to the uterus and superior to the bladder, measuring approximately 30 × 70 mm in axial diameters and characterized by uneven contrastographic impregnation with hypodense areas.

On the third day of the postpartum period, due to persistent widespread abdominal pain, abdominal tension, and a strongly positive Blumberg sign in all abdominal quadrants, a CT scan of the abdomen and pelvis with contrast medium was performed. The radiological investigation revealed a modest amount of free intra-abdominal effusion, distension of some small intestinal loops, and air-fluid levels in an occlusive context. Additionally, some small intestinal loops exhibited thickened walls, suggestive of paralytic ileus. An oval alteration within the medical umbilical ligament anterior to the uterus and superior to the bladder, characterized by uneven contrastographic impregnation with hypodense areas was demonstrated [Fig. 3].

This formation was challenging to typify but could potentially be a pedunculated myoma. Blood tests showed marked leukocytosis (WBCs: 20,900/mmc), increased CRP (201 mg/L), and procalcitonin (PCT) levels (6.6 ng/mL), elevated for the first time. The case was discussed with general surgeons, and an urgent exploratory laparoscopy was performed. Diffuse peritonitis was observed, along with a preperitoneal abscess cavity in the suprapubic median area, which drained spontaneously into the peritoneal cavity. The peritoneal fluid was sent for culture. A broad-spectrum antibiotic therapy with Piperacillin/Tazobactam, Gentamicin, and Metronidazole was initiated. Subsequently, the culture results were received: the peritoneal fluid culture showed the presence of *S. aureus*, which was sensitive to the antibiotics already being administered.

**Fig. 4:**
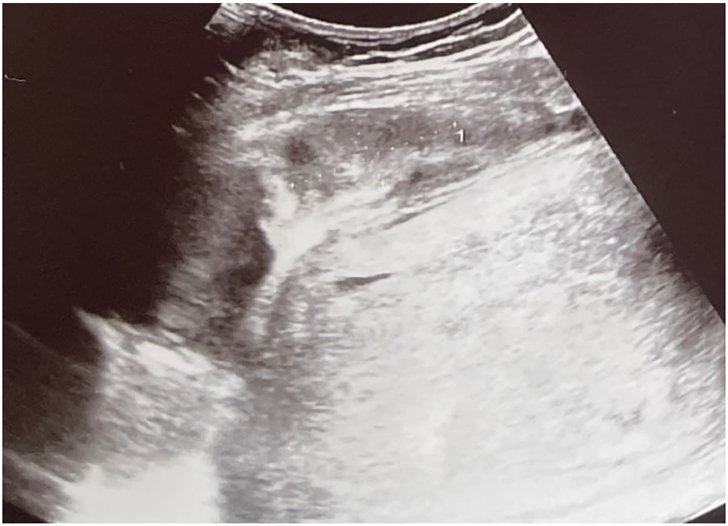
Image of an inhomogeneous lesion, with less well-defined margins compared to the previous ones, and smaller in size.

In the following days, the patient experienced fever, worsening blood test results (CRP: 350 mg/L, WBCs: 21,400/mmc initially, decreasing to 12,600), and platelet and coagulation disorders (INR: 1.5, PTT ratio: 1.31 and fibrinogen: 919 mg/dL) referable to severe undernutrition in a phase of florid sepsis. Her abdomen remained painful, with a defense reaction and a weakly positive Blumberg sign in the lower quadrants. A transabdominal ultrasound confirmed the persistence of a mass in the suprafascial, extraperitoneal area, measuring 3.7 × 3.7 × 1.8 cm, reduced in size compared to the pre-intervention exam [Fig. 4].

**Fig. 5:**
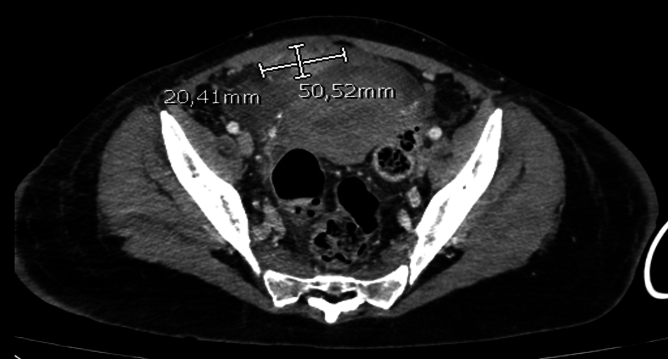
Coronal view of the CT abdomen and pelvis with IV contrast performed demonstrating a reduction in the size of the known lesion. Its maximum diameters are now approximately 50 × 20 mm.

An urgent chest and abdominal CT scan with contrast medium was repeated, showing a reduction in the size of the known collection in the lower quadrants [Fig. 5].

**Fig. 6:**
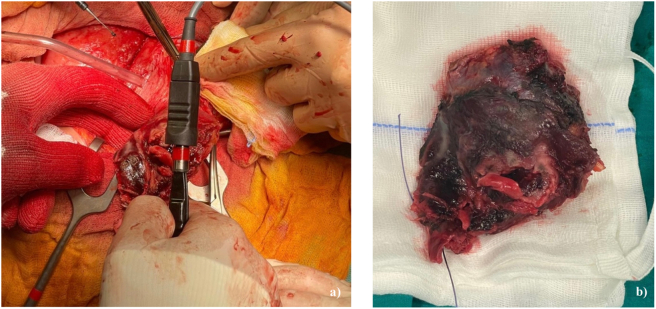
**a**) Intraoperative photo taken just before the complete removal of the abscessed urachal cyst and surrounding malacic tissue. **b)** Macroscopic image of the surgical piece.

A bilateral pleural effusion extending from the base to the lung apices, associated with a modest pleural effusion, appeared. After a discussion with general surgeons, a public laparotomy was performed. The urachus with the abscessed cyst was removed, including all surrounding inflammatory and malacic tissue [Fig. 6]. The cyst was closely related to the bladder dome but did not involve the ureters and was completely removed, sacrificing the point of contact with the bladder. The bladder solution was repaired, and the peritoneal cavity was washed and drained.

The post-operative course was complicated by progressively prolonged clotting times (INR 2.6, PTT ratio 1.5, fibrinogen 840 mg/dL, platelets 450,000/mmc on the third post-operative day) and hypoalbuminemia due to severe undernutrition during sepsis (fasting for 7 days). These issues resolved after resuming feeding and support therapy.

On the eighth day post-surgery, the bladder catheter was removed, and on the ninth day, a cystography was performed, which yielded a negative result. Consequently, the patient was discharged with complete symptom resolution and a non-painful abdomen. At discharge, her blood tests showed WBC 11,000/mmc, CRP 21 mg/dL, PCT 0.7 ng/mL, and normal coagulation.

**Fig. 7:**
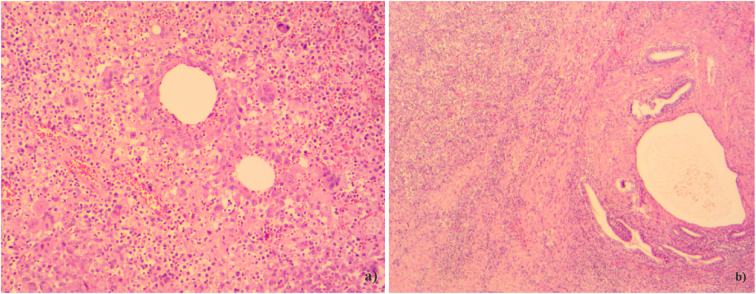
**a**) Urachal remnants appear as cystically dilated epithelial-lined structures. They are surrounded by a thin layer of fibromuscular tissue. EE, 10×. **b**) Urachal remnants with adjacent mixed inflammatory infiltrate. EE, 10×.

The final histological examination results confirmed the diagnosis of abscessed urachal cyst [Fig. 7].

## 3 Discussion

This case report enriches the few cases in the literature on the topic. The case examined is distinctive due to the rarity of abscess formation in the urachal cyst during a full-term pregnancy, and to the uncommon presentation with afebrile patient on admission.

The formation of urachal cysts is determined by the partial, or total, failure of obliteration of the channel connecting the posterior surface of the umbilical scar and the bladder dome. Consequently, while the two ends remain closed, the central lumen remains patent, forming the cyst. Typically, urachal cysts form in the lower third of the urachus and are small and asymptomatic. However, these cysts can lead to various complications, including infection with abscess formation (more common), urinary retention, bleeding, and increased incidence of urachal adenocarcinoma [2]. The luminal wall of the urachal cyst is composed of uroepithelium. The infection of the cyst may occur due to the accumulation of material inside and it can originate alternatively from i) hematogenous or lymphatic spread, ii) through direct infection of the umbilicus or iii) through direct infection of the bladder, often associated with a medical history of urinary tract infection in the previous days. Newman et al. and MacMillian et al. report *Staphylococcus aureus* as the most commonly isolated infectious agent in the endocystic fluid; other bacteria that can be isolated are both aerobic and anaerobic, such as *Escherichia coli*, *Enterococcus faecium*, and *Klebsiella pneumoniae* [5]. When infected, urachal cysts clinically present with abdominal pain, fever (up to 40 °C), dysuria - sometimes associated with a sense of incomplete bladder emptying - umbilical discharge, palpation of a midline abdominal mass, and acute abdominal crises in the case of cyst perforation with inflammation of the abdominal wall [6,7]. However, in the case report by J. Sargent, the patient was afebrile at presentation, similar to the patient of our case [8].

Hematological tests often reveal an elevated white blood cell count and CPR [2,3,7].

Due to the low incidence and heterogeneous clinical presentation, the diagnosis is often difficult and erroneous [6]. Minevich et al. reported that, in a group of 17 patients, a misdiagnosis was made in more than half of the cases [9]. Although an infected urachal cyst is rare in the adult female population, it should be considered in the differential diagnosis of acute abdomen, especially if associated with a palpable abdominal mass. The most common misdiagnoses include appendicitis, Meckel's diverticulitis, urinary tract infections, pelvic inflammatory disease, and bladder carcinoma [6]. When uncertain about the diagnosis, it is important to exercise caution in the use of prolonged antibiotic therapies as they might mask, or slow down, the diagnostic process. For instance, in our case report, the home antibiotic therapy may have contributed to a delay in the progression of the pathology and could have acted as an additional confounding factor for the diagnosis.

A woman can be asymptomatic during her entire life and only experience symptoms during pregnancy due to the unique physiologic changes in the urinary tract that occur during this period. Bladder and urethral hypotonia, along with ureteral dilation and vesicoureteral reflux, are physiological occurrences in a pregnancy. It is well-established that progesterone exerts a relaxing effect on smooth muscles and may contribute to urethral dilation, as progesterone concentration substantially increases during pregnancy. In the last weeks of pregnancy, the fetal head and the enlarging lower uterine segment occupy the true pelvis and in this way the bladder capacity becomes reduced. Urodynamic studies conducted on normal pregnancies have shown a doubling of intrinsic bladder pressure in the late pregnancy. Hence, both endocrine and mechanical factors appear essential in driving the pregnancy-related changes. It might be reliable that elevated bladder pressure, reduced capacity, and hormonal-induced dilation are enough to reopen a functionally closed patent urachus. As suggested by Dr. Gordon Peacock, bacterial proliferation is necessary to expand a partially obstructed lumen and form an infected cyst, which can open to the umbilicus, causing evident secretions, or to the bladder, or in both directions at once. The physiological alterations occurring during pregnancy can indeed significantly contribute to the cyst becoming symptomatic. Symptoms tend to intensify in late pregnancy, likely due to heightened bladder pressure resulting from fetal and uterine enlargement. Abdominal wall distention, including the umbilicus, plus the chronic urinary infection, promotes the formation of a fistula. The infection likely ascends from the urethra, as infective agents are more commonly fecal than cutaneous. During voiding, it is probable that the urachus fails to completely empty, leading to urine stagnation, which raises infection [10]. In literature there is also the hypothesis that an assisted delivery with external abdominal pressure can cause intraperitoneal rupture of the abscessed cyst [7]. In our clinical case, the patient became symptomatic at term pregnancy when abdominal distention was maximal, and she had an urinary tract infection, which likely triggered the cyst's infection.

Ultrasonography is often the initial test performed in patients with suspected urachal cyst infection [6]. Nagasaki et al. reported a diagnostic success rate of 75 % for ultrasonography, while Minevich et al. reported 57.1 %, and Cilento et al. reported 100 % [5]. Urachal cyst presents as an extra-pelvic collection of fluid with complex echogenicity, localized on the midline between the umbilicus and the bladder [2]. On ultrasound, it can be confused with an abscess, hematoma, dilated intestinal walls [10], or a fibroid, as in our case. Factors that made the ultrasonographic diagnosis challenging in our case, leading to a misdiagnosis of a colliquated fibroid, were the rarity of the event, hypoechogenicity, the presence of more anechoic areas, and well-defined margins, making it resemble the typical appearance of a fibroid. In the case report by McNally et al., a urachal duct carcinoma in a pregnant woman was also initially misdiagnosed as a degenerating fibroid. In that instance as well, the patient presented with abdominal pain and a positive urine culture, but without fever and with normal vital signs. In that case the diagnosis was possible thanks to the patient's medical history of a patent urachal duct in early life, and a second-level imaging study [11]. Computed tomography or magnetic resonance imaging (less commonly used) can be helpful in confirming, or ruling out, doubtful cases and assessing the extent of the infectious process. In a recent 2023 study by Opper Hernando et al., it is emphasized that the primary advantage of CT scan is to assist in identifying an unknown focus of sepsis. Two clinical criteria - “signs of decreased vigilance” and “increased catecholamine demand” - were deemed highly relevant for a CT request. Likewise, elevated procalcitonin and lactate levels were consistently found to be critical laboratory values to request a CT. The guidelines do not provide recommendations for the optimal time window to perform the CT scan. However, the authors suggest a time window of ≥1–6 h (but not exceeding 6 h for maximum benefits) [12]. Imaging also provides information about the cyst's size and its relationships with the bladder and surrounding tissues [6].

Complete excision of the cyst is recommended as treatment, due to the risk of malignant degeneration reaching 51 % and the rate of reinfection [2]. Two approaches are described in the literature: i) a two-step procedure involving initial incision and drainage of the abscess - accompanied by broad-spectrum antibiotic therapy - followed by elective excision, and ii) a single-step procedure involving one-time excision following antibiotic therapy [6]. The best method is still a subject of debate. Elkbuli et al. propose a brief administration of intravenous antibiotics before complete excision as a single and effective treatment for isolated urachal cyst infection [13]. However, Yoo et al., in their 2006 retrospective study comparing the two methods, identify the two-step approach as superior, associated with fewer hospitalization days (5.8 vs. 9.2 days) and no complications. In their study, some patients undergoing the single-step approach developed complications such as enterocutaneous fistula formation, requiring additional treatment [5]. However, when the cyst is perforated into the peritoneal cavity, the treatment of choice is primary excision with closure of the defect in the bladder or intestine [7]. The laparoscopic approach to urachal anomalies was first mentioned in 1993 by Trondsen et al. and is currently the preferred surgical method because it is safe, effective, and minimally invasive, thus allowing for precise tissue dissection, minimal blood loss, and faster recovery [2]. In our case, the mistake lied in not completely excising the cyst during the first laparoscopy and only draining the peritoneal abscess. This was done with the intention of performing the most conservative surgery possible, aiming to avoid continuous solutions involving the bladder. After the first procedure, the patient still had worsening inflammatory markers and only partial symptom relief. For this reason, complete excision of the lesion was essential for the patient's clinical improvement.

According to McNally et al., surgery during pregnancy is both safe and necessary in cases of severe symptoms and suspicion of malignancy. Specifically, the optimal timing for surgery is during the second trimester of pregnancy, after organogenesis is complete, and before fetal viability and significant uterine volume increase. In their case report, the patient underwent surgery at 9 weeks gestational age and at 29 weeks gestational age, successfully reaching full-term pregnancy [11].

## 4 Conclusion

This case report outlines the presentation and management of an infected maternal urachal cyst during pregnancy with diagnosis and subsequent management. While urologic pathologies are uncommon, they should be considered in the differential diagnosis for pregnant women presenting with abdominal pain and a palpable mass. Employing a multidisciplinary approach may facilitate early diagnosis and optimize clinical outcomes.

## Consent of publication

Written informed consent was obtained from the patient for publication of this case report and accompanying images. A copy of the written consent is available for review by the Editor-in-Chief of this journal on request.

## Ethical approval

Ethical approval was provided by authors' institution.

## Funding

N/A.

## Author contribution

All authors have read and approved the final version of the manuscript.

## Research registration number

N/A.

## Conflict of interest statement

The authors report no Conflict-of-Interest Statement.
